# Supplementary material for: Pyrogallol-Phloroglucinol-6,6’-Bieckol from Ecklonia cava Improved Blood Circulation in Diet-Induced Obese and Diet-Induced Hypertension Mouse Models
Source: Mar Drugs. 2019 May 8;17(5):272. doi: 10.3390/md17050272 (PMC6562948; doi:10.3390/md17050272)
Supplement: Supplementary file 1 [file marinedrugs-17-00272-s001.pdf]

Supplementary material

Table S1. List of antibodies for Immunohistochemistry, Immunofluorescence and immunoblotting (Western blot)

Antibody name	Company	Cat. No.	Immunofluorescence
ICAM-1	Invitrogen	MA5407	1:250
VCAM-1	Abcam	Ab134047	1:250
E-selectin	Santa cruz	Sc-7884	1:50
vWF	DAKO	A0082	1:250

Antibody name	Company	Cat. No.	Western blot / Immunohistochemistry
PCNA	Abcam	Ab2426	- / 1:250
Phospho-ERK (pERK)	Cell signaling	9101s	- / 1:100
Phospho-AKT (pAKT)	Santa cruz	Sc-514032	- / 1:50
Phospho-AMPK (pAMPK)	Abcam	Ab133448	- / 1:100
PI3K	Fine Test	FNab06422	- / 1:100

Table S2. List of primer for quantitative real time polymerase chain reaction (qRT-PCR)

Gene name		Primer sequence
β-actin	Forward	5'-ACAAAGCTGTTCAGTGTCTCCA-3’
	Reverse	5'-CTCCGTTTCCAGAATACACACA-3’
E-selectin	Forward	5'-ATGAAATGTCTTCCCAGTGCTT-3’
	Reverse	5'-TGATCCCTTCAGTTCAAATCCT-3’
ICAM-1	Forward	5'-ATAACCGCCAGAGAAAGATCAG-3’
	Reverse	5'-GGCTTGTCCCTTGAGTTTTATG-3’
VCAM-1	Forward	5'-GAGACCTGTCACTGTCAACTGC-3’
	Reverse	5'-CATCAGTGTAGTCTCCCCCTTC-3’
vWF	Forward	5'-GGCAAAGCATTTGTATGACTC -3’
	Reverse	5'-ATTTGTCCGTTGTCTTTCCTGT-3’
